# Detection of *Mycoplasma columbinasale* in Cases of Respiratory Disease in Domestic Pigeons (*Columba livia* var. *domestica*)

**DOI:** 10.1155/2022/3950684

**Published:** 2022-11-19

**Authors:** Giuseppe Giglia, Ilaria Porcellato, Maria Luisa Marenzoni, Elisa Rampacci, Marco Bottinelli, Andrea Matucci, Fabrizio Passamonti, Elvio Lepri

**Affiliations:** ^1^Department of Veterinary Medicine, University of Perugia, Via San Costanzo 4, 06126 Perugia, Italy; ^2^Veterinary Medicine, Utrecht University, Yalelaan 1, 3584 CL, Utrecht, Netherlands; ^3^Mycoplasma Unit-SCT1-Verona, WOAH, Reference Laboratory for Avian mycoplasmosis, Istituto Zooprofilattico Sperimentale delle Venezie, Via Bovolino 1c, 37060 Buttapietra, Italy

## Abstract

In 2017, respiratory disease and low mortality were reported in domestic flying pigeons (*Columba livia* var. *domestica*) trained as hunting live bait in a breeding farm in Umbria, Italy. Clinically, open beak breathing, dropped wings, and pharyngeal and laryngeal hyperaemia were observed. Three birds were submitted for necropsy. Gross pathological evaluation revealed in all cases diffuses hyperaemia of the tracheal mucosa in association with mild emaciation and multiorgan congestion. Microscopically, diffuse epithelial hyperplasia of the trachea (*n* = 3) and diffuse lymphocytic infiltration of the lamina propria (*n* = 3) were observed. No lesions were reported in other organs. Based on reported clinical signs and lesions, *Mycoplasma* spp. were suspected, and molecular detection was performed on tracheal specimens leading to the identification of *Mycoplasma columbinasale*. Immunohistochemistry was subsequently performed to localize the microorganism within tissue lesions. Immunohistochemistry confirmed the presence of *Mycoplasma* species on the tracheal epithelial cells of all birds. Following tylosin administration, complete resolution of the clinical condition and lack of recurrence of clinical signs were reported in the breeding farm. These findings suggest that *M. columbinasale* could potentially have a role in the respiratory disease and low mortality in domestic pigeons.

## 1. Introduction

*Mycoplasma* organisms are small bacteria lacking a cell wall. *Mycoplasma* species have been incriminated as cause of diseases in numerous animal species and can cause significant economic losses in livestock [1]. Transmission can be vertical or horizontal, the latter through airborne dust and secreted droplets [2]. In avian species, numerous *Mycoplasma* spp. (e.g., *Mycoplasma gallisepticum*, *M. synoviae*, *M. meleagridis*, and *M. iowae*) are reported as cause of diseases, especially in chickens and turkeys. In pigeons, the role of *Mycoplasma* spp. is still unclear, with descripted isolation of *M. columbinum, M. columborale,* and *M. columbinasale* in both clinically healthy birds and birds showing respiratory clinical signs [3–7]. Furthermore, reports of mycoplasma-associated disease in pigeons are few and often lack a clear association between agent and lesion. Here, we describe the detection of *Mycoplasma columbinasale* in an outbreak of respiratory disease and mortality in domestic pigeons (*Columba livia* var. *domestica*) in central Italy in 2017, describing postmortem, gross pathological, and microscopic evaluation, in association with molecular and immunohistochemical identification of the microorganism.

## 2. Case Presentation

### 2.1. Case History and Gross Post-Mortem Examination

In 2017, a breeding farm of flying pigeons (*Columba livia* var. *domestica*), trained as hunting live bait, reported signs of respiratory disease in 10% of birds, in association with low mortality (3%) and overall poor performance, in Umbria (Italy). Animals were vaccinated against *Avian Paramyxovirus* type 1 (APMV-1). Clinically, birds were showing open beak breathing, increased respiratory effort, wheezing, and dropped wings. Signs were waxing and waning following long-term unsuccessful treatment cycles with sulphonamides, nitroimidazoles (Sulfamethoxazole Trimethoprim and dimetridazole), and quinolones (Enrofloxacin 10%). During clinical examination, the clinician reported diffuse and moderate pharyngeal and laryngeal hyperaemia in association with the previously mentioned signs. Nestlings were not affected, while first signs of disease were developed after weaning, concurrently with the initial phase of training. Three spontaneously dead birds with respiratory signs were submitted to the Veterinary Pathology Diagnostic Service of the Department of Veterinary Medicine in Perugia (Italy) for postmortem investigation. Complete necropsies were performed with concurrent gross examination. In all three examined animals, subtle gross changes were observed. Mild emaciation with reduced fat deposits and mild bilateral pectoral muscle atrophy were identified. A mild and diffuse dark-red discolouration and organomegaly were seen in liver and spleen (congestion). A diffuse light-red discolouration was noticed in the lung and tracheal mucosa, the latter also showing a diffuse, mild to moderate, increased thickness. No other lesions or changes were observed in other organs.

### 2.2. Histology and Immunohistochemistry

**Figure 1:**
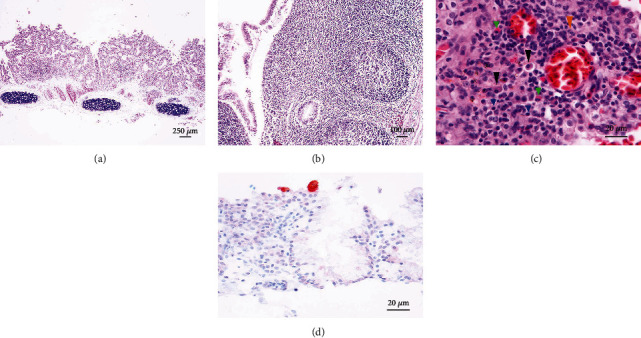
Histology and immunohistochemical detection of *Mycoplasma* spp. in domestic pigeons. (a) Trachea (H&E); marked epithelial hyperplasia and increased cellularity of the lamina propria and multifocal areas of erosion and ulceration. (b) Trachea (H&E); submucosal lymphoid aggregates. (c) Lung (H&E); mononuclear aggregates are characterized by small lymphocytes (short blue arrowhead), plasma cells (long black arrowhead), macrophages (long orange arrowhead), and occasional heterophils (short green arrowhead). (d) Trachea (IHC); *Mycoplasma* spp. seen as finely granular intracytoplasmic reaction on the respiratory epithelium.

Tissues from all organs were sampled for pathology and immersion-fixed in 10% neutral-buffered formalin during necropsy, or stored at -20°C, for subsequent molecular testing. For histology, formalin-fixed tissues were embedded in paraffin and, subsequently, 5 *μ*m sections were cut and stained with routine Haematoxylin and Eosin stain. For the immunohistochemistry, briefly, 5 *μ*m sections were cut from the three cases and mounted on poly-L-lysine-coated slides. After dewaxing and rehydration, slides were pretreated in a microwave with Tris-EDTA buffer (pH 9.0) for 20 minutes and, after cooling, washed with phosphate-buffered saline (PBS) and incubated with a mouse monoclonal antibody pan-avian *mycoplasma* MAB-IZSVE-PANMYC-137 (cross-reactive with different avian *mycoplasma* species) diluted 1 : 500 for 2 hours at room temperature. After Tris-buffered saline (TBS) washing, slides were treated with a diluted biotinylated secondary antibody for 30′; subsequently, ABC ready-to-use kit (Abcam, Cambridge, UK) was used according to the manufacturer's instructions. Positive reaction was revealed with 3-amino-9-ethilcarbazole (Abcam). Mayer's haematoxylin was applied as a counterstain. Negative controls were incubated with TBS, omitting the primary antibody. Ovine *Mycoplasma* sp. PCR-positive lungs with lesions compatible with mycoplasma-associated disease were used as positive control. On histology, in all birds, the most striking lesion was identified in the trachea, which showed a diffuse and marked epithelial hyperplasia, in association with loss of cilia and multifocal areas of erosion (Figure 1(a)). A moderate and diffuse inflammatory infiltrate was expanding an oedematous lamina propria. The inflammatory infiltrate was characterized by a high number of small mature lymphocytes, with occasional histiocytes and rare plasma cells. Multifocally, submucosal lymphoid aggregates were prominent (Figure 1(b)). Moderate changes were also noticed on the vessels of the lamina propria, showing moderate ectasia with a high number of circulating erythrocytes (hyperaemia). The bronchial lamina propria of the lungs showed diffuse and marked accumulation of amorphous weakly eosinophilic to basophilic material (oedema). Multifocal lymphoid aggregates of varying size as well as the infiltration of a moderate number of small lymphocytes, occasional mature heterophils, and rare histiocytes were also present in one case (Figure 1(c)). The surrounding pulmonary parenchyma showed diffuse and moderate hyperaemia and aerial capillaries with the occasional presence of oedema. Only in one case was noticed a focal area characterized by loss of the tissue architecture and replacement with eosinophilic amorphous material mixed with pyknotic nuclei and cellular debris (colliquative necrosis), surrounded by numerous macrophages, epithelioid cells and in smaller numbers, multinucleated giant cells of the “foreign body” type (granuloma). In the air sacs of all cases, occasional small mature lymphocytes and histiocytes infiltrating the stroma were observed, while the lining epithelium appeared multifocally, mildly hyperplastic. Of the other organs, only the liver showed a diffuse and mild ectasia of sinusoids with an increase in the number of circulating erythrocytes (congestion), and in one case, in the periportal space, extramedullary haematopoiesis was also noticed. No additional lesions were observed in other organs. On immunohistochemistry, immunolabelling for *Mycoplasma* was visualized as a finely granular intracytoplasmic reaction on the respiratory epithelium (Figure 1(d)).

### 2.3. Molecular Detection and Genome Sequencing

Tracheal and cloacal swabs were obtained using sterile cotton swabs, rotated against the mucosa of trachea or cloaca and kept in tubes containing 0.5 mL of PBS, pH 7.2. Two hundred *μ*L of transport buffer (PBS) of the swabs or 20 mg of lung and trachea was used for DNA extraction using the commercial kits QIAamp DNA Mini kit (QIAGEN) and GenElute Mammalian Genomic DNA Miniprep kit, (Merck, Italy) for swabs and tissues, respectively, according to the manufacturer's instructions.

A conventional PCR, targeting a fragment of 1013 bp of the 16S rRNA gene, was used to detect *Mycoplasma* spp. (Lierz et al. 2007). A nested PCR protocol targeting the 16S rRNA gene was used to detect *Chlamydia* spp. (Messmer *et al.* 1997). This method discriminates *Chlamydia. pneumoniae* from *Chlamydia. psittaci* amplifying fragments of 221 or 127 bp, respectively. PCR products of the expected size were purified using a commercial kit (Qiaquick, Qiagen, Germany) and directly submitted for sequencing on both strands with the same forward and reverse primers (Biofab srl, Italy). By PCR, *Mycoplasma* spp. DNA was amplified from the tracheal swabs and tracheal tissue of the tested animals, while no DNA amplification was detected in cloacal swabs and lung tissues. Amplicon sequences were assembled using BioEdit (version 7.2) [8]. Assembled sequences were aligned with nucleotide database available from GenBank using the basic local alignment search tool (BLAST) (https://blast.ncbi.nlm.nih.gov/Blast.cgi) to find similarities with *Mycoplasma* sequences. Amplicons were sequenced and BLAST analysis resulted more than 99% identity with the homologous 16S rRNA gene sequences of *Mycoplasma columbinasale*. The sequences were submitted to GenBank and assigned the accession numbers MW732628 and MW732629. All the specimens (tracheal swab, cloacal swab, lung and trachea) tested negative for *Chlamydia* sp. by nested PCR.

### 2.4. Microbiological Examination

Microbiological assessment was performed on lung tissues and tracheal swabs collected during post-mortem examination. Multiple lung specimens were individually homogenized in sterile PBS. One-hundred microliters of these and tracheal swabs were spread on 5% defibrinated sheep blood agar, MacConkey agar and Mannitol salt agar (Liofilchem, Italy). The plates were incubated at 37°C in aerobic conditions and 5-10% CO_2_ for 24-48 h. The identification of bacteria was performed by examining colony characteristics, Gram staining, and use of biochemical tests and commercially available Analytical Profile Index (API) kits (bioMerieux, Étoile, France). The samples were cultured also on Sabouraud dextrose agar (Liofilchem, Italy) supplemented with and without chloramphenicol at 28 and 35°C for up to 7 days for fungal examination. Tracheal swabs from both pigeons grew sporadic mixed cultures containing from two to three different bacterial species. Bacteria were isolated as scanty colonies and belonged to genus *Cronobacter*, *Corynebacterium*, *Enterococcus* and *Bacillus* with no predominating organism. Fungi were not detected from tracheal swabs. Lung specimens were negative for bacteria and fungi.

### 2.5. Treatment and Clinical Follow-Up

Tylosin was administered subcutaneously to birds with respiratory signs (BID for 5 days; 25 mg/kg). For clinically healthy birds in close aviaries, administration was set as 5 gr of water soluble tylosin/L drinking water for 7 days, as reported in the leaflet. Birds were clinically monitored for the entire length of the treatment and routine monitoring of the healthy status of the breeding aviary is performed yearly. The treatment with tylosin successfully led to the complete resolution of the clinical condition. Lack of recurrence of clinical signs was reported in the breeding aviary in the following years.

## 3. Discussion and Conclusion

In this study, molecular detection and immunohistochemistry were used to detect and identify *Mycoplasma* spp. in tissues of domestic pigeons (*Columba livia* var. *domestica*) submitted for postmortem investigations during an episode of respiratory disease and low birds' mortality in a pigeon breeding farm in central Italy. Complete necropsy and gross and histopathological investigation were performed in association with the previously mentioned ancillary diagnostic tests.

Some *Mycoplasma* spp. organisms are known pathogens in birds, causing every year severe economic loss in the poultry industry [9]. In pigeons, the role of these biological agents is not completely understood. Few reports of mycoplasma-associated disease are available in literature, often lacking a clear agent-lesions association. Additionally, the isolation of the same agents in healthy birds is a confounding factor for cause-and-effect relationship [3, 4, 6, 7].

In this study, a diffuse tracheal hyperaemia with moderate mucosal thickening was noticed as a major finding in association with nonspecific splenic-hepatomegaly and diffuse multiorgan congestion. On histological examination, the most striking lesions were the epithelial hyperplasia associated with a moderate to severe lymphohistiocytic tracheitis. Epithelial hyperplasia is commonly reported in mycoplasma-associated disease in domestic and wild avian species [10, 11]. Particularly in birds, the same pattern of lesions has been described in *M. gallisepticum*, naturally infected house finches (*Carpodacus mexicanus*), and experimentally infected domestic canaries (*Serinus canaria domestica*) [11, 12], and the increased tracheal mucosal thickness is usually adopted for *M. gallisepticum* disease severity measurement [13, 14]. Regarding as the core of disease pathogenesis, *M. gallisepticum* attachment to the host cell elicits a sturdy lymphoproliferative host immune response which is responsible for tissue damage (immunopathology) at the colonization site [15, 16].

On immunohistochemistry, specific *Mycoplasma* labelling was seen on the apical surface of the tracheal respiratory epithelium as reported in previous papers, confirming the presence of the microorganism in close association with the lesions [17]. Therefore, immunohistochemistry performed with pan-avian *mycoplasma* monoclonal antibody proved itself useful for localizing the *Mycoplasma* within the tissue section, hence helping the achievement of the diagnosis.

Regarding the molecular detection and genomic sequencing, although the identification of *M. columbinasale* has been reported in healthy pigeons raising awareness on its possible role as commensal and part of the normal respiratory microbiome of pigeons [5], the association with the previously mentioned data supports its role as agent of disease, as proposed in another report [7].

The role of *M. columbinasale* in the development of the previously described lesions and clinical signs was also suspected and based on the results of microbiology testing. A mixed culture with scarce no predominating organisms that are not usually pathogens of avian upper respiratory tract [18] is less likely to produce the observed disease.

A possible limitation of this study is the lack of *Mycoplasma* spp. cultivation which is performed in specialised laboratories, and it is known for the tediousness and the difficulties that come with it. Also, the sensitivity of *Mycoplasma* cultivation may be variable due to low bacterial load and copresence of different *Mycoplasma* species with different growing requirements [19, 20].

In addition, this study lacks of data regarding possible concurrent coinfections (e.g., *Pigeon circovirus* (PiCV)) that could have contributed to the development of an immune suppressive state with consequent establishment of *M. columbinasale* as an opportunistic pathogen [5, 21, 22]. Although histological lesions found in this case are similar to what can be observed during *M. gallisepticum* infection, those alone do not allow proposing analogies with the pathogenesis of this major avian mycoplasmal pathogen. Additionally, it is not known if *M. columbinasale* is able to exert an immunosuppressive effect in the same way as *M. gallisepticum* does [23–25]. Taken into account that clinical signs were observed in adult birds only, it is possible that the physical activity performed by this category of pigeons may have predisposed them to disease development. Further studies are needed to better understand any interrelation between *M. columbinasale* and the pigeon immune system.

Finally, the complete resolution of the clinical condition in absence of recurrence of clinical signs was obtained the administration of tylosin. The successful outcome of the treatment further supported, as “ex-juvantibus diagnosis”, the possible involvement of *M. columbinasale* in the respiratory disease.

In conclusion, the association of clinical signs, gross and histologic lesions, molecular detection of *M. columbinasale* and its immunohistochemical identification in lesions, and the clinical resolution with administration of tylosin suggest for this agent its possible association to respiratory disease, poor performance and low mortality in domestic pigeons.

## Data Availability

Sequences are available on GenBank with assigned accession numbers MW732628 and MW732629.
